# Long term exposure to L-arginine accelerates endothelial cell senescence through arginase-II and S6K1 signaling

**DOI:** 10.18632/aging.100663

**Published:** 2014-05-17

**Authors:** Yuyani Xiong, Michael Forbiteh Fru, Yi Yu, Jean-Pierre Montani, Xiu-Fen Ming, Zhihong Yang

**Affiliations:** Vascular Biology, Department of Medicine, Division of Physiology, University of Fribourg, CH-1700, Fribourg Switzerland

**Keywords:** Arginase-II, endothelium, L-arginine, Senescence, mTOR, adhesion molecules

## Abstract

L-arginine supplementation is proposed to improve health status or as adjunct therapy for diseases including cardiovascular diseases. However, controversial results and even detrimental effects of L-arginine supplementation are reported. We investigate potential mechanisms of L-arginine-induced detrimental effects on vascular endothelial cells. Human endothelial cells were exposed to a physiological (0.1 mmol/L) or pharmacological (0.5 mmol/L) concentration of L-arginine for 30 minutes (acute) or 7 days (chronic). The effects of L-arginine supplementation on endothelial senescence phenotype, i.e., levels of senescence-associated beta-galactosidase, expression of vascular cell adhesion molecule-1 and intercellular adhesion molecule-1, eNOS-uncoupling, arginase-II expression/activity, and mTORC1-S6K1 activity were analyzed. While acute L-arginine treatment enhances endothelial NO production accompanied with superoxide production and activation of S6K1 but no up-regulation of arginase-II, chronic L-arginine supplementation causes endothelial senescence, up-regulation of the adhesion molecule expression, and eNOS-uncoupling (decreased NO and enhanced superoxide production), which are associated with S6K1 activation and up-regulation of arginase-II. Silencing either S6K1 or arginase-II inhibits up-regulation/activation of each other, prevents endothelial dysfunction, adhesion molecule expression, and senescence under the chronic L-arginine supplementation condition. These results demonstrate that S6K1 and arginase-II form a positive circuit mediating the detrimental effects of chronic L-arginine supplementation on endothelial cells.

## INTRODUCTION

L-arginine is a semi-essential amino acid which is not only involved in protein synthesis, is also a single substrate for endothelial nitric oxide synthase (eNOS) to produce the important vasoprotective molecule nitric oxide (NO) [1, 2]. Depletion of L-arginine causes eNOS dysfunction in cultured endothelial cells [3]. Hence, L-arginine supplementation has been widely used under many physiological and pathological conditions aiming to improve health status or to treat diseases including cardiovascular diseases [2, 4-7]. However, controversial results have been reported. Although many studies demonstrate that acute or short-term supplementation of L-arginine improves endothelium-dependent vaso-dilation or reduces blood pressure in diseased animal models or patients with cardiovascular diseases [8-15], numerous studies with L-arginine supplementation, however, show no sustained effects on endothelial function [16-20]. Most importantly, studies with long-term (6 months) L-arginine supplementation even show harmful effects in atherosclerotic animal models [21] as well as in patients with cardiovascular diseases for unknown reasons [22, 23]. It seems that the impact of L-arginine on cardiovascular function depends on duration of the amino acid supplementation.

The harmful effects of chronic L-arginine supplementation have been recently recapitalized on vascular endothelial cells by Scalera and colleagues [24]. They showed that chronic L-arginine supplementation is capable of accelerating endothelial cell senescence associated with enhanced expression of arginase-II (Arg-II) and decreased endothelial NO generation. This detrimental effect of L-arginine is prevented by Arg-II gene silencing, suggesting that chronic L-arginine supplementation causes endothelial dysfunction through up-regulation of Arg-II, an enzyme that metabolizes L-arginine and is predominantly involved in accelerating vascular endothelial cell senescence [25]. The mechanism of Arg-II gene up-regulation by L-arginine, however, remains unknown. Our recent study demonstrates that a persistent activation of the mammaliantargetofrapamycincomplex-1 (mTORC1) and its down-stream target S6K1 promotes endothelial senescence and dysfunction through up-regulation of Arg-II [25].

Given that mTORC1-S6K1 pathway can be activated by nutritional components including amino acids, which occurs through the Leucyl-tRNA synthetase-Rag [26, 27], we hypothesize that chronic L-arginine supplementation may cause endothelial dysfunction and senescence through mTORC1-S6K1 pathway and Arg-II.

## RESULTS

### Chronic, but not acute L-arginine treatment promotes endothelial senescence phenotype

**Figure 1:**
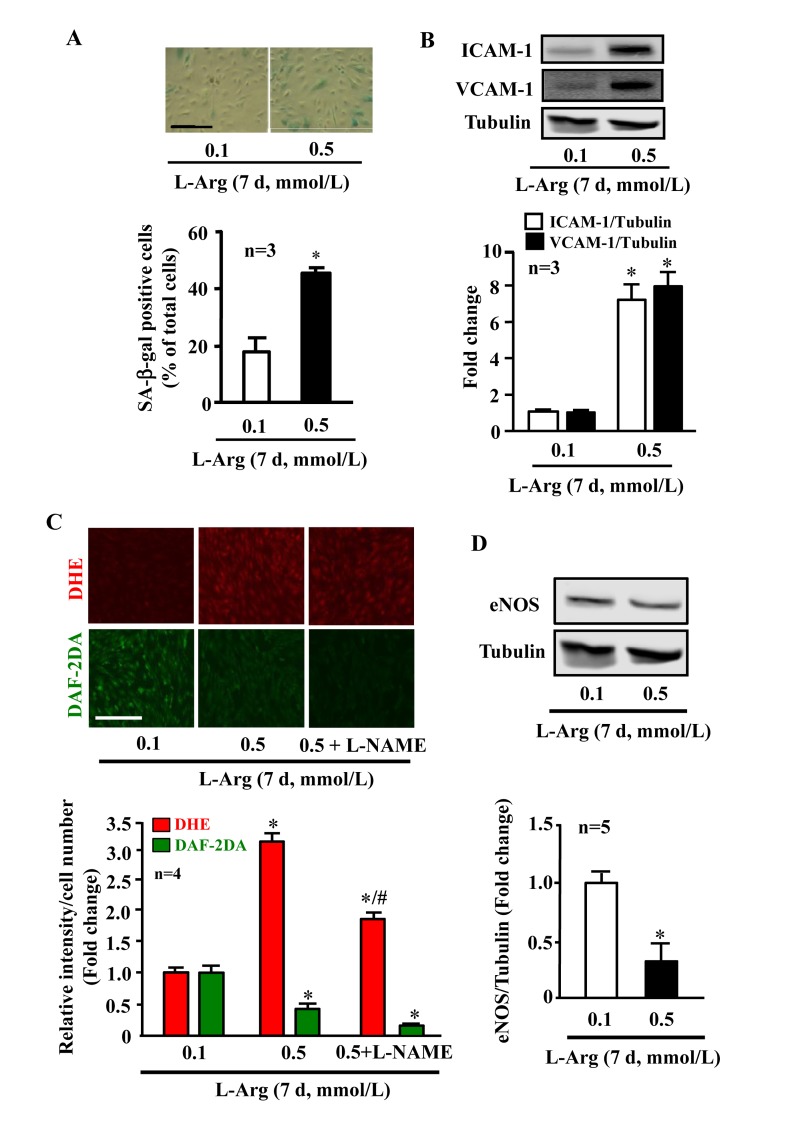
Chronic L-arginine supplementation promotes endothelial senescence, inflammation and eNOS-uncoupling. Confluent young HUVECs were L-arginine-starved overnight followed by treatment with 0.1 mmol/L or 0.5 mmol/L L-arginine for 7 days (7 d) with change of medium every 48 hours. Cells were serum starved overnight prior to experiment. **(A)**SA-β gal assay. **(B)**Western blot analysis of ICAM-1/VCAM-1. Tubulin served as loading control. **(C)** Detection of O_2_^.-^ and NO by DHE and DAF-2DA staining, respectively. The eNOS inhibitor L-NAME (1 mmol/L) was added to the cells 2 hours prior to the DAF-2DA staining. **(D)** Western blot analysis of eNOS protein levels with tubulin as loading control. The Western blot analysis of expression of ICAM-1, VCAM-1 and eNOS shown in (B) and (D), respectively, was performed with the same membrane. The quantification of the signals is presented as graphics below the corresponding images or blots. n represents the number of the repeated independent experiments. *p < 0.05 vs 0.1 mmol/L; #*p* < 0.05 vs 0.5 mmol/L. Scale bar = 200 μm

**Figure 2:**
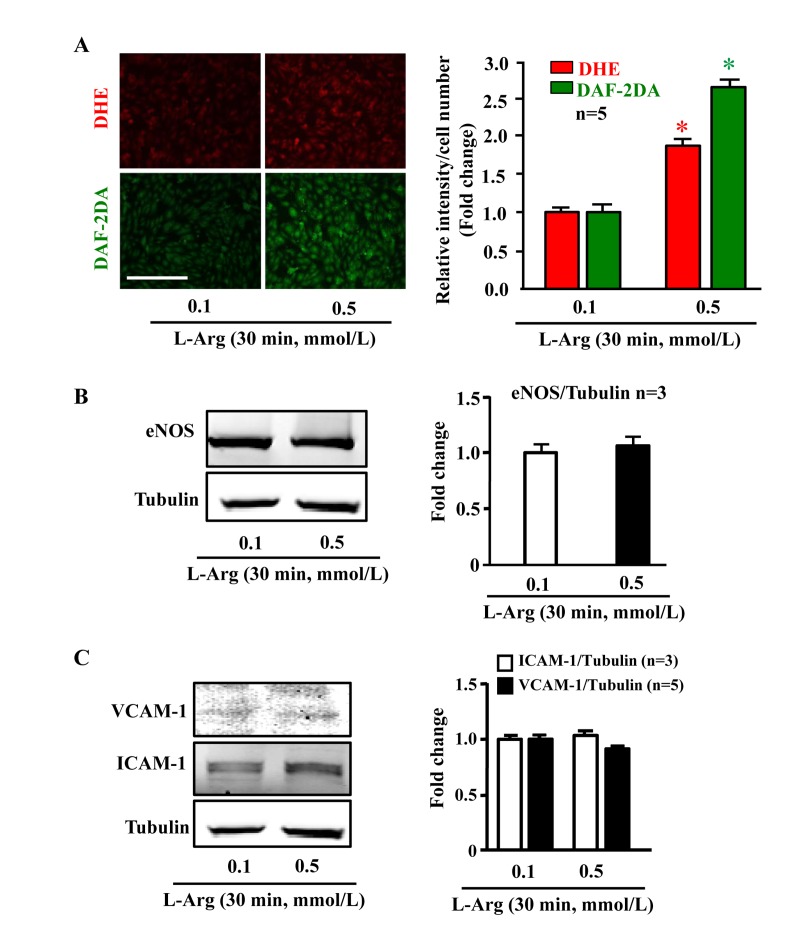
Acute L-arginine supplementation enhances production of both NO and superoxide anion Cells were serum- and L-arginine-starved overnight followed by treatment with 0.1 mmol/L or 0.5 mmol/L L-arginine for 30 minutes. (**A**) Detection of O_2_^.−^ and NO by DHE and DAF-2DA staining, respectively. (**B**) Western blot analysis of eNOS protein level with tubulin as loading control. (**C**) Western blot analysis of ICAM-1/VCAM-1 levels. The Western blot analysis shown in (**B**) and (**C**) was performed with the same membrane. Tubulin served as loading control. The quantification of the signals is presented as graphics at the corresponding right panels. *p < 0.05 vs 0.1 mmol/L. Scale bar = 200 μm

In young endothelial cells, when compared to cells treated with the physiological concentration of L-arginine (0.1 mmol/L), chronic treatment of the cells with a higher concentration of L-arginine (0.5 mmol/L) for 7 days enhanced the number of SA-β-gal positive cells (Fig. 1A), protein levels of intercellular adhesion molecule-1 (ICAM-1) and vascular cell adhesion molecule-1 (VCAM-1) (Fig. 1B). Moreover, treatment of the cells with L-arginine at the concentration 0.5 mmol/L for 7 days caused endothelial dysfunction as measured by enhanced superoxide anion and decreased NO production (Fig. 1C). Inhibition of eNOS by L-NAME (1 mmol/L for 2 hours) abolished NO production as expected, but also reduced superoxide anion levels, demonstrating eNOS-uncoupling under the chronic L-arginine treatment (Fig. 1C). In addition, chronic treatment of the endothelial cells with L-arginine (0.5 mmol/L, 7 days) significantly decreased eNOS protein levels (Fig. 1D). In contrast to the chronic L-arginine treatment, acute treatment of the cells with L-arginine at the same concentration (0.5 mmol/L) for 30 minutes enhanced NO production but also increased superoxide anion generation while having no effects on protein levels of eNOS, VCAM-1 and ICAM-1 (Fig. 2).

### Chronic L-arginine treatment increases Arg-II and mTORC1/S6K1 signaling in endothelial cells, while acute treatment activates mTORC1/S6K1 without increase in Arg-II

**Figure 3:**
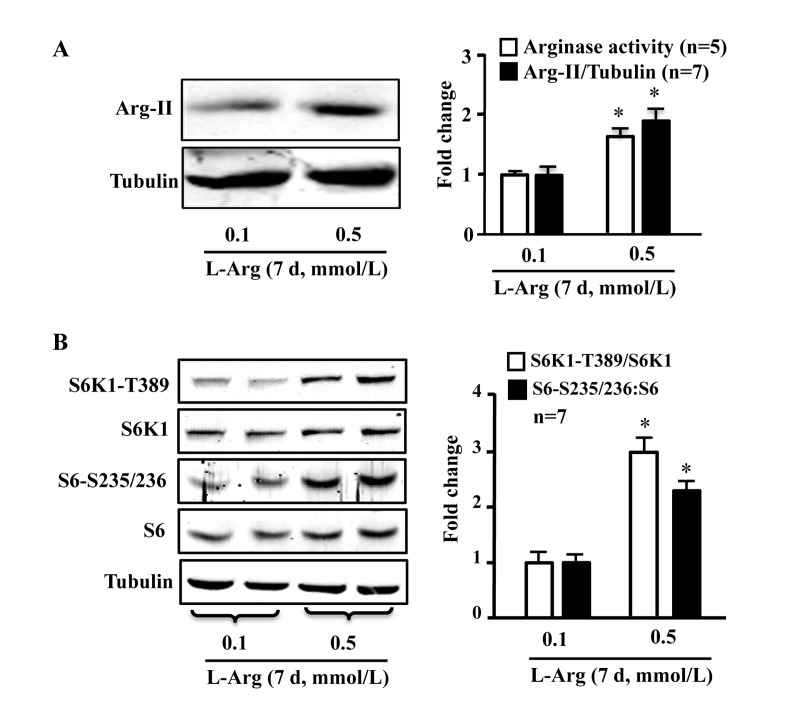
Chronic L-arginine supplementation induces Arg-II expression/activity and mTORC1-S6K1 signaling Cells were treated as described in Fig.1. **(A)** Western blot analysis of Arg-II expression and arginase activity assay. **(B)** Western blots showing activation of mTORC1 and S6K1 as monitored by phosphorylation of S6K1-T389 and S6-S235/236, respectively. The graphics at the corresponding right panels present the quantification of the signals. *p < 0.05 vs 0.1 mmol/L

**Figure 4:**
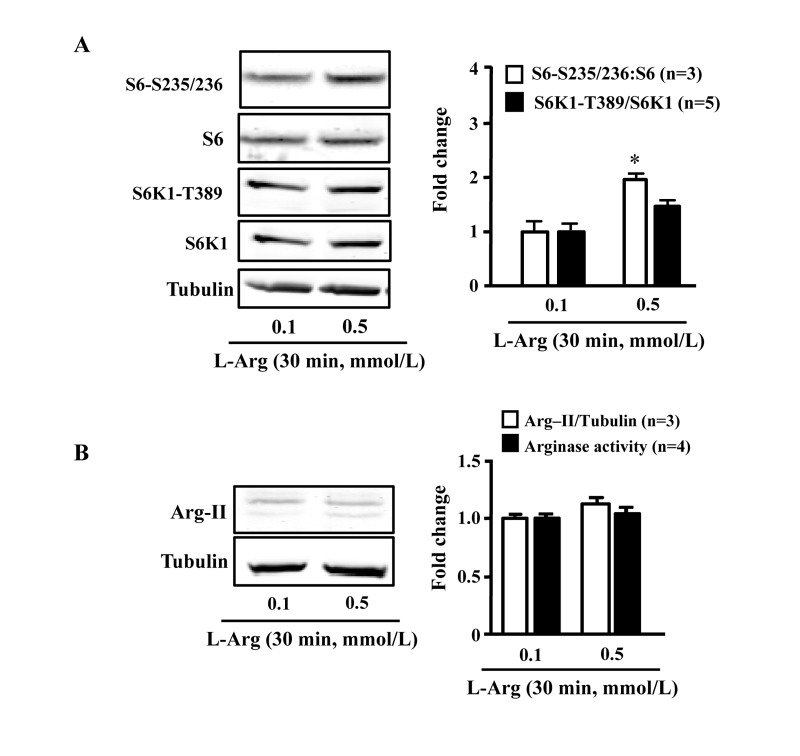
Acute L-arginine supplementation activates mTORC1-S6K1 signaling, but not Arg-II expression/activity Endothelial cells were treated as described in Fig. 2. (**A**) Western Blots showing activation of mTORC1 and S6K1 as monitored by phosphorylation of S6K1-T389 and S6-S235/236, respectively. (**B**) Western Blot analysis of Arg-II expression and arginase activity assay. The graphics at the corresponding right panels present the quantification of the signals. *p < 0.05 vs 0.1 mmol/L

In young endothelial cells, chronic L-arginine treatment (0.5 mmol/L, 7 days) enhanced Arg-II expression and activity (Fig. 3A) as well as activation of mTORC1/S6K1 signaling as measured by enhanced ratio of S6K1-T389/S6K1 and that of S6-S235/236:S6, when compared to cells treated with 0.1 mmol/L of L-arginine (Fig. 3B). In contrast to chronic treatment, acute treatment of the cells with L-arginine (0.5 mmol//L) for 30 minutes enhanced mTORC1-S6K1 signaling without having an effect on Arg-II expression or activity (Fig. 4).

### Positive crosstalk between Arg-II and mTORC1/S6K1 mediates the chronic effect of L-arginine on cell senescence

**Figure 5:**
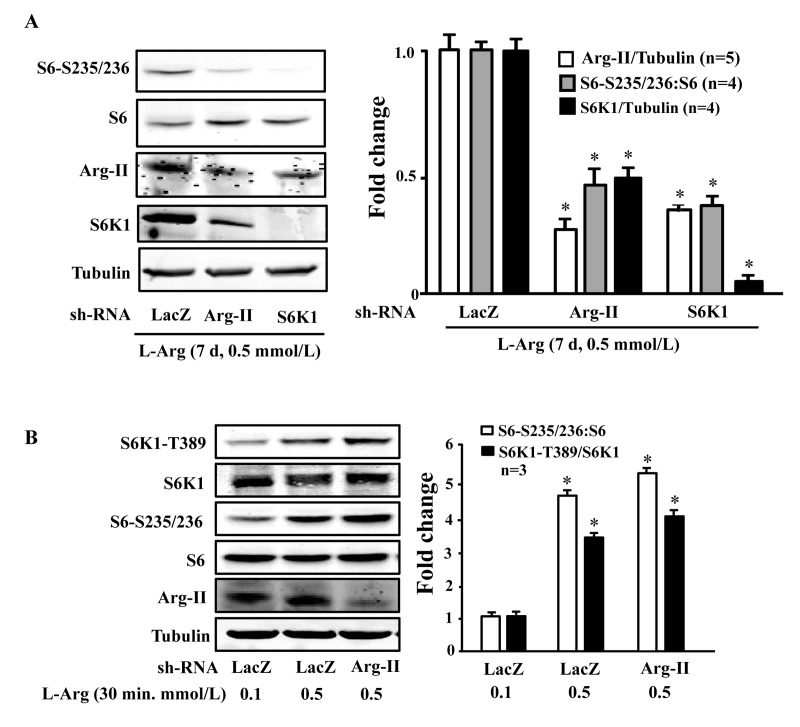
S6K1 and Arg-II form a positive regulatory circuit under the condition of chronic L-arginine supplementation (**A**) Endothelial cells were treated as described in Fig. 1, except that the cells were transduced with the rAd/U6-LacZ^shRNA^ as control, rAd/U6-Arg-II^shRNA^ or -S6K1^shRNA^ after L-arginine-starvation and prior to the treatment with only 0.5 mmol/L of L-arginine for 7 days. Shown are Western blot analyses of S6-S235/236, total S6, Arg-II and S6K1, and the quantification of the signals. *p < 0.05 vs rAd/U6-LacZ^shRNA^+0.5 mmol/L L-arginine control. **(B)** Endothelial cells were treated as described in Fig. 1, except that the cells were transduced with the rAd/U6-LacZ^shRNA^ as control or rAd/U6-Arg-II^shRNA^48 hours prior to serum- and L-arginine-starvation. Shown are Western blot analyses of S6K1-T389, S6K1, S6-S235/236, total S6, Arg-II, tubulin, and the quantification of the signals. *p < 0.05 vs rAd/U6-LacZ^shRNA^+0.1 mmol/L L-arginine

To evaluate the role of Arg-II and S6K1 in the chronic effects of L-arginine on cell senescence, Arg-II and S6K1 were knocked down by transducing the cells with rAd/U6-shRNA against Arg-II or S6K1, after overnight L-arginine-starvation and prior to addition of L-arginine 0.1 or 0.5 mmol/L, 7 days). The up-regulation of Arg-II and sustained activation of mTORC1-S6K1 pathway by chronic treatment of L-arginine (0.5 mmol/L, 7 days) depended on each other, as silencing either Arg-II or S6K1 prevented up-regulation/activation of the other (Fig. 5A). Since S6K1 is activated by acute treatment of L-arginine (0.5 mmol/L, 30 minutes) without Arg-II up-regulation (Fig. 4), we wish to determine whether the basal Arg-II level is required for L-arginine-induced acute S6K1 activation. As revealed by Fig. 5B, silencing Arg-II did not affect the S6K1 activation by acute L-arginine treatment (0.5 mmol/L, 30 minutes).

**Figure 6:**
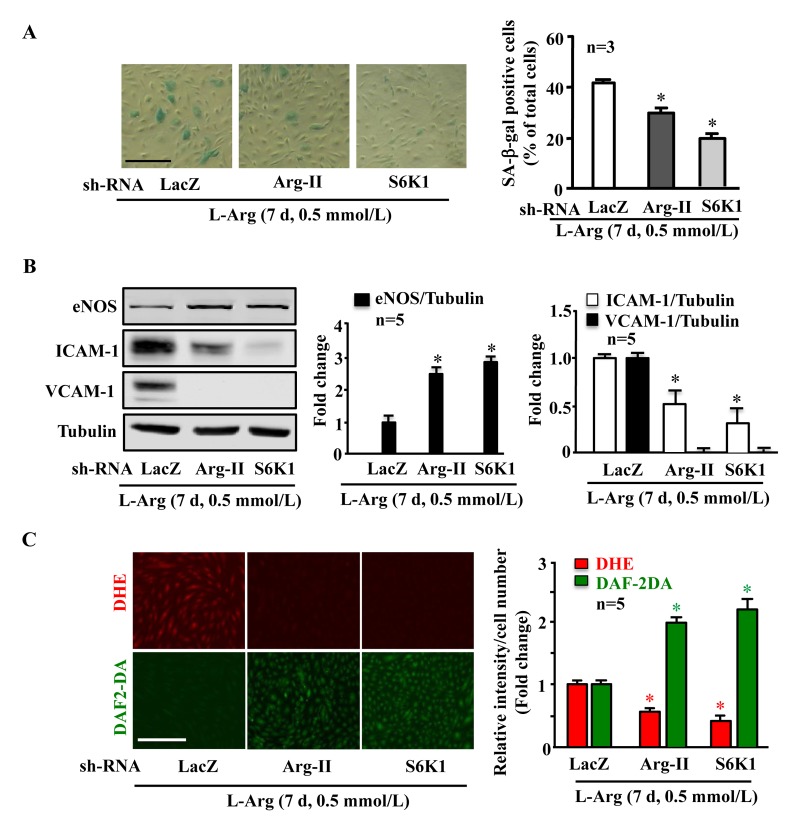
Arg-II and S6K1 mediate the chronic effect of L-arginine on cell senescence Endothelial cells were treated as in Fig. 5. (**A**) SA-β gal assay. (**B**) Western blot analysis of eNOS, ICAM-1, VCAM-1. Tubulin served as loading control. (**C**) DHE staining for detection of O_2_^.−^, DAF-2DA staining for detection of NO. The graphics at the corresponding right panels present the quantification of the signals. *p < 0.05 vs rAd/U6-LacZshRNA control+0.5 mmol/L L-arginine. Scale bar = 200 μm

**Figure 7:**
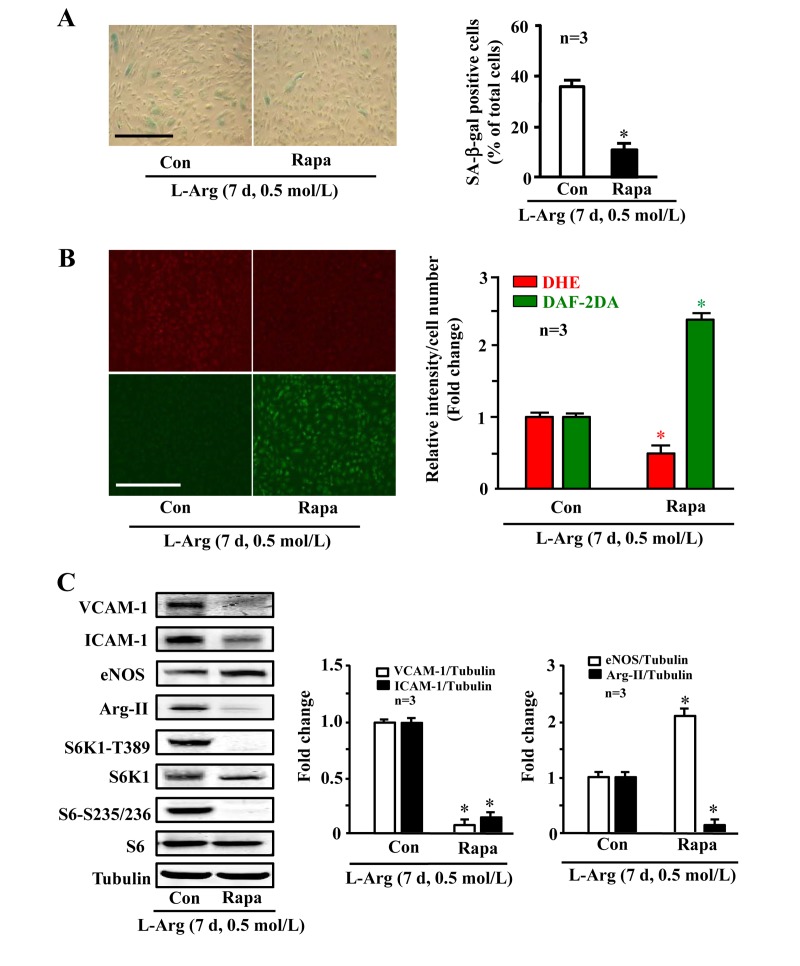
Arg-II and S6K1 mediate the chronic effect of L-arginine on cell senescence Endothelial cells were treated as in Fig. 1 except that rapamycin (20 ng/ml) was added at same time as L-arginine (0.5 mmol/L). **(A)** SA-β gal assay. **(B)** DHE staining for detection of O_2_^.−^, DAF-2DA staining for detection of NO. **(C)** Western blot analysis of VCAM-1, ICAM-1, eNOS, Arg-II, S6K1-T389, S6K1, S6-S235/236 and S6. Tubulin served as loading control. The bar graphs are the quantifications of signals from the corresponding experiments shown in the left panels. *p < 0.05 vs L-arginine alone as the control (Con). Scale bar = 200 μm

Along with the observation presented in Fig. 5A, silencing either Arg-II or S6K1 significantly reduced the SA-β-gal positive cell number (Fig. 6A), enhanced eNOS protein level while decreasing ICAM-1 and VCAM-1 levels (Fig. 6B), inhibited superoxide anion and enhanced NO production (Fig. 6C) under condition of chronic L-arginine treatment (0.5 mmol/L, 7 days). Similar results were obtained when cells were treated with the mTORC1 inhibitor rapamycin. As shown in Fig. 7, the cell senescence induced by chronic L-arginine treatment (0.5 mmol/L, 7 days) as analyzed by the positive SA-β-gal cells was significantly reduced by rapamycin (20 ng/ml, 7 days, Fig. 7A). Moreover, rapamycin (20 ng/ml, 7 days) reduced superoxide anion production (DHE staining) and increased NO generation (DAF-2DA staining, Fig. 7B), inhibited expression levels of VCAM-1, ICAM-1, Arg-II, while enhanced eNOS levels in the endothelial cells under the condition of chronic exposure to L-arginine (0.5 mmol/L, 7 days, Fig. 7C). As expected, activation of S6K1 signaling (measured by S6K1-T389 phosphorylation levels and S6-S235/236 levels) was inhibited by rapamycin under this condition (Fig. 7C).

## DISCUSSION

Since discovery that L-arginine is the only substrate for endothelial cells to produce the vasoprotective molecule NO via eNOS [3], L-arginine supplementation is widely investigated for decades to boost NO production in different models including human subjects in vivo. However, results from paramount numbers of published studies in the literature are very confusing. Beneficial, no sustained effects, and harmful effects including enhanced mortality rate in clinical studies have been reported with L-arginine supplementation therapy [28]. Importantly, a randomized, double-blinded, placebo-controlled study in patients with acute myocardial infarction, the VINTAGEMI study, demonstrates that 6-month oral L-arginine supplementation (3 g 3 times a day on top of standard post infarction therapy) does not have any benefits on vascular stiffness and left ventricular ejection fraction, but increases mortality [22]. A second clinical trial in patients with peripheral artery disease, the so-called NO-PAIN study with the same protocol, shows decreased NO production and shortened walking distance in patients receiving L-arginine supplementation as compared to the placebo group [23]. The impact of L-arginine supplementation, particularly chronic supplementation for treatment of cardiovascular diseases seems detrimental. However, the mechanisms underlying these findings remain obscure. A recent study in cultured cells suggests that Arg-II could be involved in the detrimental effects of chronic L-arginine supplementation [24]. This group shows that chronic L-arginine supplementation causes endothelial senescence which can be prevented by silencing Arg-II gene [24].

Arg-II has been shown to play an important role in various diseases including atherosclerosis, type-II diabetes and vascular aging [25, 28-31]. The capability of L-arginine to induce Arg-II expression was first shown by Crody et. al. in 1989 in the renal epithelial HEK cell line [32]. Our present study further confirms this result and demonstrates that supplementation of L-arginine to endothelial cells for 7 days causes Arg-II up-regulation along with endothelial senescence phenotype as demonstrated by increased SA-β-gal staining, inflammatory adhesion molecule expression, eNOS-uncoupling, i.e., decreased NO production and enhanced superoxide anion generation which is sensitive at least in part to the eNOS inhibitor L-NAME. Our results are fully in line with the findings by Scalera and colleagues [24]. Moreover, our study has explored the underlying mechanisms of the detrimental effects of chronic L-arginine-induced endothelial Arg-II up-regulation in the endothelial cells and demonstrates that this is mediated by mTORC1-S6K1. Among other regulators, amino acids are potent activators of mTORC1-S6K1 pathway [33]. Indeed, we show that in endothelial cells, L-arginine at a modest concentration 0.5 mmol/L significantly activates mTORC1/S6K1 over 7 days as compared to a lower concentration 0.1 mmol/L, which is associated with increased Arg-II up-regulation and activity. Silencing S6K1 not only inhibits Arg-II up-regulation, but also prevents endothelial cell senescence, enhancement of adhesion molecule expression, as well as eNOS-uncoupling under chronic L-arginine supplementation condition, demonstrating the important role of mTORC1-S6K1 in up-regulation of Arg-II and endothelial senescence. This conclusion is further supported by the experiment showing that pharmacological inhibition of mTORC1-S6K1 signaling with rapamycin reveals the same effects as S6K1 silencing. Our results are in line with the concept that mTORC1-S6K1 pathway is involved in loss of regenerative/replicative potential and drives geroconversion, a conversion from reversible cell cycle arrest to irreversible cell senescence [34-39].

Our previous study showed a positive crosstalk between S6K1 and Arg-II in the replicative endothelial cell senescence [25]. Here, we show that this crosstalk also exists in L-arginine-induced cell senescence, since silencing either S6K1 or Arg-II inhibits the activity of each other and is able to prevent endothelial dysfunction, inflammation and senescence. Taking into account that acute L-arginine-treatment (30 minutes) causes S6K1 activation without having any changes in Arg-II protein level and that silencing Arg-II does not affect S6K1 activation by the acute L-arginine treatment (30 minutes), it is likely that initial acute S6K1 activation by L-arginine, which is mediated by Leucyl-tRNA - Rag GTPase [26, 27], does not require Arg-II, but leads to up-regulation of Arg-II. The enhanced Arg-II in turn positively modulates mTORC1-S6K1 activity resulting in sustained activation of S6K1, which may involve either inhibition of negative regulatory mechanisms of mTORC1-S6K1 pathways or activation of positive regulators of the signaling pathway. The detailed mechanisms of the positive regulatory circuit between S6K1 and Arg-II remain to be investigated.

There are studies demonstrating that NO production from eNOS is primarily determined by extracellular L-arginine bioavailability which directly channels to the cell membrane-associated eNOS [40-43]. Our present study with acute L-arginine supplementation further supports this concept by showing that short term L-arginine supplementation for 30 minutes enhances NO production. As expected, acute L-arginine supplementation does not alter eNOS expression, levels of adhesion molecules and Arg-II. It is, however, accompanied with superoxide anion generation, which obviously does not decrease the bioavailability of NO. The L-arginine-induced superoxide generation may be linked to acute activation of mTORC1/S6K1 pathway which is capable to produce the free radical from mitochondrion [44, 45]. If this signaling is persistently activated such as under the condition of chronic L-arginine supplementation, an oxidative stress, i.e., eNOS-uncoupling then occurs as shown in our present study. It is obvious that endothelial dysfunction under chronic L-arginine supplementation could not be attributed to L-arginine deficiency, because there is a constant concentration of L-arginine (0.5 mmol/L) available in the culture medium. We cannot exclude the possibility that intracellular specific L-arginine pool is depleted because of up-regulation of Arg-II. However, this concept is not supported by studies showing that continuous cellular exposure to L-arginine for 7 days does not affect L-arginine uptake and generation of the endogenous eNOS inhibitor ADMA [46]. Our previous study showed that Arg-II causes eNOS-uncoupling requiring L-arginine metabolizing enzymatic activity [25]. Taking into account of a recent study demonstrating that the metabolic products of arginase such as urea and L-ornithine, or downstream enzymes do not contribute to eNOS inhibition [47], it still remains elusive how Arg-II causes eNOS-uncoupling in an enzymatic activity-dependent manner.

In conclusion, our present study provides evidence demonstrating that L-arginine exerts either beneficial or detrimental effects on endothelial cells which is dependent on the duration of the supplementation. We show that short term L-arginine supplementation enhances endothelial NO production, while long-term supplementation causes endothelial senescence through stimulation of mTORC1-S6K1 signaling and up-regulation of Arg-II. The findings may not only explain the contradictory or inconsistent results on the vascular effects of L-arginine supplementation reported in the literature, but also provide the mechanistic insight into the detrimental clinical outcomes in patients with cardiovascular diseases. The L-arginine supplementation in combination of mTORC1-S6K1 inhibition may preserve the beneficial effect on NO production while diminishing the detrimental effects of chronic L-arginine supplementation on endothelium, which may represent a novel therapeutic option for L-arginine supplementation therapy.

## MATERIALS AND METHODS

All chemicals, if not specifically indicated, including those used for Western blotting, mouse anti-tubulin monoclonal antibodies were obtained from Sigma (Buchs, Switzerland). Rabbit antibodies against phospho-S6-S235/236 (#2211s), S6 (#2217s) and anti-VCAM-1 (#12367S) were from Cell Signaling (Allschwil, Switzerland); mouse anti-eNOS (#610297) and anti-S6K1 (#611260) were from BD Transduction laboratories (Allschwil, Switzerland); rabbit anti-Arg-II (sc-20151) and mouse anti-ICAM-1 (sc-8439) were purchased from Santa-Cruz Biotech (Nunningen, Switzerland); Alexa Fluor680-conjugated anti-mouse IgG (A21057) and dihydroethidium (DHE) were from Molecular Probes/Invitrogen (Lucerne, Switzerland); X-gal was from Promega (Dübendorf, Switzerland); the membrane-permeable 4,5-diaminofluoresceine acetate (DAF-2DA) was from VWR international SA (Dietikon, Switzerland); QuickTiter™ Adenovirus Titer Immunoassay Kit (VPK-109, Cell Biolabs, Inc) were purchased from LuBioScience GmbH (Luzern, Switzerland); endothelial cell growth supplement (ECGS) pack was from PromoCell GmbH (Allschwil, Switzerland); all cell culture media and materials were purchased from Gibco BRL (Lucerne, Switzerland).

### Recombinant adenovirus (rAd)

Generation of rAd expressing shRNA targeting human Arg-II and S6K1 driven by the U6 promoter (rAd/U6-hArg-II^shRNA^and -hS6K1^shRNA^, respectively) was described previously [48] The control rAd expressing shRNA targeting LacZ (rAd/U6-LacZ^shRNA^) was from Invitrogen life Technologies. The rAd titer (infectious unit: ifu/ml) was determined by staining the largest and most abundant structural proteins “hexon proteins” in the adenovirus capsid using QuickTiter™ Adenovirus Titer Immunoassay Kit after infecting HER911 cells, derived from human retina cells by Adenovirus E1 transformation [49], for 48 hours.

### Cell culture and adenoviral transduction of the cells

Human umbilical vein endothelial cells (HUVECs) [50] were cultured at 37°C and 5% CO_2_ to confluency using RPMI-1640 containing 5% fetal calf serum (FCS), ECGS, Amphotericin B and antibiotics (penicillin and streptomycin). Cells of passage 1 to 3 (P1 to P3)were used as young cells which were further split in a ratio of 1:3 continuously over a period of several weeks till replicative senescence was reached as assessed by senescence-associated (SA)-β-galactosidase (SA-β-gal) staining [25]. For the acute L-arginine treatment experiments, cells were serum- and L-arginine-starved overnight followed by treatment of the cells with physiological (0.1 mmol/L) or high concentrations (0.5 mmol/L) of L-arginine for 30 minutes. For the long-term/chronic L-arginine supplementation studies, cells were cultured overnight in L-arginine-free RPMI-1640 medium containing 5% FCS and ECGS followed by culture in the above medium supplemented with 0.1 mmol/L or 0.5 mmol/L of L-arginine for 7 days. The medium was changed every 48 hours. Before harvesting, cells were serum starved overnight. For the recombinant adenovirus-mediated silencing experiments, the protocol was the same as the long-term treatment, except that the transduction of the cells with rAd at ~200 multiplicities of infection (MOI) was performed after L-arginine-starvation and prior to the treatment with only 0.5 mmol/L of L-arginine for 7 days.

### Western blot

Cell lysate preparation, SDS-PAGE and Western blot, antibody incubation and signal detection were carried out as described previously [30]. Quantification of signals was performed using NIH Image J (NIH, Bethesda, MD, USA).

### Arginase activity assay

Arginase activity in cells lysates was measured by colorimetric determination of urea formed from L-arginine as described previously [31].

### Activity of mTORC1 and S6K1

mTORC1 and S6K1 activity were analyzed by monitoring phosphorylation of S6K1 at threonine-389 (S6K1-T389) and its substrate S6 at serine-235/serine-236 (S235/S236), respectively, by Western blotting.

### Senescence-associated β-galactosidase (SA-β-gal) staining

Cells were initially washed twice with PBS followed by fixation with 3.7% formaldehyde solution in PBS for 10–15 min. After washing twice with PBS, cells were then incubated with the SA-β-gal staining solution (1 mg/ml X-gal, 40 mmol/L citric acid, 5 mmol/L potassium ferrocyanide, 5 mmol/L potassium ferricyanide, 150 mmol/L sodium chloride, 2 mmol/L magnesium chloride dissolved in phosphate buffer, pH 6.0) for 16 hours at 37°C in a CO_2_-free atmosphere. The stained senescent cells were detected by conventional microscopy.

### Detection of nitric oxide (NO) and superoxide levels in cultured endothelial cells

For detection of NO, HUVECs were gently washed twice with Ca^2+^-free PBS, and incubated in a modified Krebs-Ringer bicarbonate solution (in mmol/L, NaCl 118, KCl 4.7, CaCl_2_ 2.5, MgSO_4_ 1.2, KH_2_PO_4_ 1.2, NaHCO_3_ 25, EDTA 0.026, and glucose 5.5) containing 5 μmol/L of DAF-2DA for 30 minutes. For measurement of superoxide, cells were incubated with 5 μmol/L DHE in culture medium for 20 minutes. For functional eNOS-uncoupling study, cells were pre-treated with the eNOS inhibitor L-NAME (1 mmol/L) for 2 hours before DHE was added. After staining, the cells were washed three times and images were obtained with Zeiss fluorescence microscopy. The intensity of the fluorescence was quantified by Image J software (U. S. National Institutes of Health).

### Statistics

Data are given as mean±SEM. In all experiments, n represents the number of independent sets of experiments. Statistical analysis was performed with Student's unpaired t-test or analysis of variance (ANOVA) with Bonferroni post test. Differences in mean values were considered significant at two tailed p ≤ 0.05.
